# Fatal *Toxoplasma gondii* COUG strain infections in southern sea otters (*Enhydra lutris nereis*): New insight on contributing factors and parasite serotyping

**DOI:** 10.1371/journal.pone.0332223

**Published:** 2025-09-10

**Authors:** Devinn M. Sinnott, Melissa Miller, David Arranz-Solís, Felipe Rodriguez, Jeroen P. J. Saeij, Francesca Batac, Katherine Greenwald, Colleen Young, Michael D. Harris, Mary Gomes, Karen Shapiro

**Affiliations:** 1 Department of Pathology, Microbiology, and Immunology, School of Veterinary Medicine, University of California, Davis, California, United States of America; 2 Marine Wildlife Veterinary Care and Research Center, California Department of Fish and Wildlife, Santa Cruz, California, United States of America; 3 Karen C. Drayer Wildlife Health Center, School of Veterinary Medicine, University of California, Davis, California, United States of America; 4 SALUVET, Animal Health Department, Faculty of Veterinary Sciences, Complutense University of Madrid, Madrid, Spain; Beni Suef University Faculty of Veterinary Medicine, EGYPT

## Abstract

Fatal infections with the rare COUG strain of the zoonotic parasite *Toxoplasma gondii* were recently detected for the first time in four southern sea otters (*Enhydra lutris nereis*) exhibiting severe protozoal steatitis. The objectives of this study were to describe new COUG strain infections in sea otters, investigate the potential contributory role of a recently discovered parasite-infecting narnavirus (*Apocryptovirus odysseus*) in these infections, assess the potential contribution of vitamin E deficiency in the development of systemic steatitis, and explore the utility of serotyping for strain-specific diagnosis of *T. gondii* infections in sea otters. Since initial reporting, six additional sea otters died due to fatal COUG strain *T. gondii* infections. Five animals exhibited lesion patterns resembling the prior case definition including severe, widespread steatitis. The final case died due to severe *T. gondii*-associated meningoencephalitis with no grossly or microscopically apparent steatitis. In contrast with a recent report utilizing a cougar-derived parasite isolate, *A. odysseus* RNA was not detected in sea otter-derived COUG strain isolates, suggesting that this narnavirus is not associated with fatal COUG strain infections in sea otters. Serotyping using dense granule (GRA) peptides to distinguish between *T. gondii* strains infecting sea otters suggests that Type X, Type II, and COUG strains exhibit different peptide-reactivity profiles that may allow them to be distinguished serologically. COUG strain *T. gondii* infections are an emerging threat to southern sea otter population health, and this strain has the potential to infect other animal and human hosts that share their environment and food sources with sea otters. Additional studies are needed to clarify the environmental sources, epidemiology, pathophysiology, and premortem serodiagnosis of COUG strain *T. gondii* infections in southern sea otters and other susceptible hosts.

## Introduction

*Toxoplasma gondii* is a globally distributed, zoonotic protozoal pathogen that commonly infects southern sea otters (*Enhydra lutris nereis*) along the central coast of California with an infection prevalence of 62% [1]. Infection with *T. gondii* in southern sea otters ranges from subclinical to severe, fatal disease [2]. Fatal toxoplasmosis is an important cause of mortality for this federally-listed threatened host species, serving as a primary or contributory cause of death for 11% of sea otters examined in one recent study [1]. Domestic and wild felids serve as definitive hosts for *T. gondii* and shed infective oocysts into the environment in their feces [3,4]. Any warm-blooded species, including humans, can serve as intermediate hosts. After ingestion of sporulated oocysts by intermediate hosts, rapidly multiplying tachyzoites disseminate throughout the body and eventually form quiescent bradyzoites within the central nervous system, heart, skeletal muscles, and other tissues as host immunity develops. Death can occur during the acute, systemic phase of infection or from chronic meningoencephalitis due to recrudescence of latent bradyzoites in the brain [5].

An unusual manifestation of fatal toxoplasmosis was first detected in southern sea otters in 2020, followed by three additional cases in 2022. This case series described four sea otters that died from acute systemic toxoplasmosis characterized by grossly and histologically striking protozoa-associated steatitis affecting all subcutaneous and internal adipose tissues with only mild inflammation in the brain [6]. Steatitis due to *T. gondii* infection had never been reported in over 20 years of southern sea otter disease surveillance. Molecular characterization of *T. gondii* DNA extracted from brain, adipose, and/or parasites isolated in culture revealed that all four cases were infected with the COUG strain of *T. gondii*. Detection of the COUG strain was surprising given that only Type X, Type II, or variants and recombinants of these strain types had ever been previously identified in sea otters, with only Type X and X variant strains associated with fatal toxoplasmosis [6]. The COUG strain is highly virulent in rodent models [7,8], and the ability of this strain to cause acute infection and death in adult, otherwise healthy individuals suggests that this strain also exhibits high virulence in southern sea otters [9].

The COUG and RUB strains are unique among *T. gondii* genotypes in that they elicit a type I interferon response in murine macrophages and human fibroblasts. It is hypothesized that this atypical immune response may contribute to the enhanced virulence of these strains in in-vitro models [8]. A novel parasite-infecting narnavirus, *Apocryptovirus odysseus*, was recently discovered in the original cougar-derived COUG strain isolate (TgCgCa1) that has been maintained in culture since 1995, as well as a human-derived RUB strain isolate [10]. Infection of protozoal isolates with this narnavirus may modulate the host immune system and shift it to an antiviral, type I interferon response that could contribute to the hypervirulence exhibited by these strains in in-vitro and rodent models [10]. The prevalence of narnavirus infection in other COUG strain isolates, including those infecting sea otters, and the role of this virus in the pathogenesis of naturally occurring COUG strain infections is unknown.

The affinity of the COUG strain for affecting adipose tissue is not fully understood. The close association between *T. gondii* organisms and steatitis lesions in sea otters suggests that the inflammation is being driven by the parasites [9]. However, other common causes of steatitis such as vitamin E deficiency, which causes steatitis in mink and other mustelids [11], have not been investigated as potential contributing factors in the pathogenesis of this lesion in sea otters.

Given the high virulence of COUG strain *T. gondii* infections in this vulnerable sea otter population, rapid diagnosis of this newly recognized form of toxoplasmosis is needed. The ability to serologically detect infection with the COUG strain in live sea otters would provide a valuable premortem clinical diagnostic tool. Serotyping assays using an array of *T. gondii* dense granule (GRA) peptides have been developed to serologically distinguish between infection with different *T. gondii* strains in humans, rodent models, and domestic livestock species [12–14]. The application and optimization of GRA peptides in sea otters may similarly allow for the serological distinction between the COUG strain and other *T. gondii* strains infecting sea otters.

The objectives of this study were to: 1) report and describe additional cases of *T. gondii* COUG strain infections in sea otters since 2022; 2) test for *A. odysseus* infection in sea otter-derived COUG strain isolates; 3) assess the possible contribution of vitamin E deficiency in the development of systemic steatitis; and 4) use GRA peptides to identify a unique seroreactivity profile that can distinguish infection with the COUG strain from other *T. gondii* strains in sea otters.

## Materials and methods

### Study animals and ethics statement

Southern sea otters examined in this study included animals that stranded dead or moribund and were humanely euthanized by the California Department of Fish and Wildlife (CDFW) in accordance with Section 109(h) of the U.S. Marine Mammal Protection Act (MMPA) and the U.S. Fish and Wildlife Service’s regulations implementing the MMPA at 50 CFR 18.22(a), and in accordance with the Service’s regulations implementing the U.S. Endangered Species Act at 50 CFR 17.21(c)(3). These statutes allow state wildlife agencies, including CDFW, to intervene on behalf of free-ranging marine mammals for their protection and welfare, including providing humane euthanasia. As a state wildlife agency, CDFW operates under these federal statutes using standardized animal care protocols with veterinary oversight for sea otter assessment and euthanasia. CDFW as a state agency is not required to have an institutional review board or ethics committee. A gross necropsy was performed for all sea otters by veterinary pathologists and other CDFW staff. Tissue samples, serum, and pericardial fluid were collected from fresh or previously frozen/thawed, necropsied sea otter carcasses.

### Histopathology and cause of death determination

Tissues selected for histopathologic examination varied depending on the postmortem condition of individual sea otters. At minimum, a partial set of tissues including brain, heart, tongue, liver, lung, spleen, and adipose tissue were examined microscopically for all sea otters. For freshly euthanized cases or animals found dead within a short postmortem interval, a more extensive set of tissues was examined microscopically (e.g., multiple sections of brain, kidney, gastrointestinal and reproductive tracts, lymph nodes, etc.). Tissues were fixed in 10% neutral buffered formalin, trimmed, embedded in paraffin, sectioned at 5 µm thickness, and stained with hematoxylin and eosin (H&E). Histopathology slides were reviewed by at least one veterinary pathologist (MM, DMS) for evidence of *T. gondii* infection, with particular examination of internal and subcutaneous adipose tissue. Features examined included: 1) the presence and relative burden (low, medium, high) of various *T. gondii* stages (tachyzoites, tissue cysts) in affected tissues, and 2) the presence, relative severity (mild, moderate, severe), and type (lymphoplasmacytic, granulomatous, etc.) of inflammation associated with protozoal organisms in affected tissues. Evidence of comorbidities or other concurrent disease processes were also noted. A ranked list of up to four causes of death (one primary cause and up to three contributory causes) was determined for each sea otter.

### Parasite isolation

The COUG strain was isolated from brain tissue of one sea otter in the previously published case series (Case 2) [9]. For one sea otter that was freshly deceased at the time of necropsy in the present study (Case 10), isolation of parasites from brain tissue was performed as previously described [9]. Briefly, brain tissue was collected aseptically and placed in antibiotic/antifungal saline for at least 24 hours at 4°C. The tissue was homogenized and approximately 1 ml of homogenized tissue was added to 10 ml of trypsin-EDTA (0.25%), incubated at 37°C for one hour, centrifuged, then added to a flask of MA-104 monkey kidney cells. The flask was incubated at 37°C and 5% CO_2_ for two hours, after which the flask was rinsed with media to remove the homogenized tissue. Cultures were maintained at 37°C and 5% CO_2_ for 30 days and monitored three times per week for signs of parasite growth, at which time fresh media was exchanged. Once parasite growth was well established, supernatant containing extracellular parasites was collected and centrifuged to pellet tachyzoites for subsequent DNA extraction.

### DNA extraction and polymerase chain reaction (PCR) targeting the ITS1 locus

Depending on samples available for each case, DNA was extracted from frozen brain, adipose tissue, and/or pelleted cultured parasites using a DNeasy Blood and Tissue kit (Qiagen, Valencia, CA) according to the manufacturer’s protocol with one modification: 180 µl ATL buffer and 30 µl proteinase K were added to each sample to pre-incubate at 56°C overnight. Each sample was screened for protozoa using a nested PCR assay targeting the multi-copy ITS1 locus as previously described [15]. Each internal PCR product was electrophoresed on a 2% agarose gel stained with RedSafe (Bulldog Bio, Portsmouth, NH) or GelGreen (Biotium, San Francisco, CA) and visualized with an ultraviolet transilluminator. Positive bands were excised and purified using a QIAquick gel extraction kit (Qiagen, Valencia, CA) according to the manufacturer’s protocol. Purified amplicons were submitted for sequencing in forward and reverse directions (UC Davis DNA Sequencing Facility, Davis, CA, or Genewiz, South San Francisco, CA). Forward and reverse sequences were trimmed and aligned (Geneious, version 11.1.5, Auckland, New Zealand) and consensus sequences were compared to reference sequences in GenBank using BLAST (https://blast.ncbi.nlm.nih.gov/Blast.cgi).

### *Toxoplasma gondii* genotype characterization

Each sample that was confirmed as containing *T. gondii* DNA via screening at the ITS1 locus was further characterized using a multilocus sequence typing (MLST) approach targeting 13 loci: B1, SAG1, 5’SAG2, 3’SAG2, altSAG2, SAG3, BTUB, GRA6, C22-8, C29-2, L358, PKI, and Apico. Amplification at the B1 locus was performed using a nested PCR assay as previously described [16]. Amplification of the remaining 12 loci was performed using a nested, multiplex assay as previously described [17]. If amplification was not successful with the multiplex assay, a nested simplex assay was performed using the same primers and cycling conditions. Gel electrophoresis, gel purification, and sequencing were performed as described for the ITS1 assay. Sequences were trimmed and compared with known reference sequences for Type I, Type II, Type III, Type X, and COUG strains at each locus using local alignment in Geneious.

### RNA extraction, cDNA synthesis, and reverse transcription (RT)-PCR for *A. odysseus*

For two otters in which the COUG strain was isolated from brain tissue in culture (Cases 2 and 10), RNA was extracted from pelleted tachyzoites using a RNeasy Plus Mini Kit (Qiagen, Valencia, CA) and cDNA was synthesized using a Reliance Select cDNA Synthesis Kit (Bio-Rad, Hercules, CA) according to the manufacturer’s instructions. Amplification of the RUB/COUG strain of *A. odysseus* was performed as previously described using cDNA from the two sea otter-derived COUG isolates, an RH (Type I) strain isolate (negative control), and the cougar-derived TgCgCa1 COUG strain isolate (positive control) [10]. The same reaction without reverse transcriptase was performed for each sample as a negative control for genomic DNA contamination and to rule out *A. odysseus* as an endogenous viral element. Amplification of GRA1 mRNA was simultaneously performed to assess the quality of cDNA as previously described [10].

### Vitamin E testing

To rule out the possibility of vitamin E deficiency as a cause of (or contributor to) steatitis, frozen liver samples from three sea otters with COUG strain-associated systemic steatitis and two age- and sex-matched control sea otters that lacked gross and histologic evidence of steatitis were submitted for evaluation of vitamin E levels via high performance-liquid chromatography with fluorescent detection (California Animal Health and Food Safety Laboratory, Davis, CA). Selected animals were those that had at least 10 g of frozen liver tissue available for testing and an estimated postmortem interval of <24 hours.

### Serotyping ELISA

Serum or pericardial fluid from COUG strain-infected sea otters (n = 7), as well as otters previously confirmed as being infected with Type X (n = 9) or Type II (n = 9) strains [7], were used to evaluate a serotyping assay designed to distinguish between the major *T. gondii* strains infecting sea otters. Sea otters infected with Type X variants or recombinants were excluded from this study. An indirect enzyme-linked immunosorbent assay (ELISA) was performed using the following nine GRA peptides which were previously shown to have the greatest utility in distinguishing between Types I, II, and III strains of *T. gondii* in human, rodent, and domestic animal serum: GRA6-I/III-213, GRA6-II-214, GRA3-I/III-43, GRA3-II-43, GRA6-I-44, GRA6-II-44, GRA6-III-44, GRA7-II-224, and GRA7-III-224 [13, 14]. All peptides were coupled to a Keyhole-Limpet Hemocyanin (KLH) carrier protein (Biomatik USA, Wilmington, DE). Lyophilized peptides were resuspended in MilliQ water or glacial acetic acid (0.1–1%) to a final concentration of 1 µg/µl. A whole parasite lysate mixture of homogenized *T. gondii* tachyzoites representing RH and GT1 (Type I), PRU, ME49, and M4 (Type II), and VEG and CEP (Type III) strains [13] served as a positive control for each assay to ensure reactivity of the samples. A peptide derived from a randomized GRA6 sequence coupled to KLH [18] was used in each assay as a negative control. Serum from a sea otter confirmed to be infected with *T. gondii* via PCR and histopathology with a *T. gondii* titer of >1:40,960 (positive) as determined by an indirect fluorescent antibody test (IFAT) [19] served as a positive control for each assay. Serum from a sea otter that had no evidence of *T. gondii* infection histologically and was negative for *T. gondii* by PCR in brain, heart, and tongue with a *T. gondii* titer of 1:40 (negative) as determined by IFAT served as a negative control for each assay.

Each peptide stock was diluted in sodium bicarbonate buffer (100mM, pH 9.6) to a concentration of 0.01 µg/ul and 100 µl of diluted peptide was added to wells (1 µg/well) of a 96-well high-binding microtiter plate. For each assay, 0.1 µg/well of *T. gondii* lysate was added as a positive control and 1 µg/well KLH-coupled control peptide was added as a negative control. Plates were covered and incubated overnight at 4°C. Plates were then rinsed three times with 0.1% PBS-Tween-20 (PBS-T) and non-specific binding was blocked by adding 200 µl of 2% milk diluted in PBS-T to each well followed by incubation at room temperature for two hours. A 1:400 dilution of each sea otter serum or pericardial fluid was prepared in 2% milk. Plates were rinsed with PBS-T and 100 µl of diluted serum or pericardial fluid was added to each well followed by incubation at room temperature for one hour. A 1:10,000 dilution of horseradish peroxidase (HRP)-conjugated goat 𝛼-ferret IgG antibody (ab112770, Abcam) was prepared in 2% milk. Plates were rinsed five times with PBS-T and 100 µl of diluted secondary antibody was added to each well followed by incubation at room temperature for one hour. Plates were rinsed five times in PBS-T and 100 µl of ABTS substrate (Roche, Mannheim, Germany) was added to each well followed by incubation at room temperature in the dark. Optical density (OD) values were measured at 405 nm (OD_405_) every five minutes. The reaction was stopped by adding 100 µl of 0.3M oxalic acid to each well when the lysate OD_405_ of the positive control serum sample reached 1.5–1.8 (typically 20–40 minutes). Final OD_405_ values were read after the reaction was stopped.

To normalize OD_405_ values for each sample and peptide, a relative index percent (RIPC) was calculated for each sample-peptide pair using the following equation: *RIPC = ((peptide OD*_*405*_ *of sample – peptide OD*_*405*_ *of negative control)/(lysate OD*_*405*_ *of sample – lysate OD*_*405*_ *of negative control)) x 100*. A ratio of RIPC values for homologous peptides (e.g., GRA6-I/III-213 vs. GRA6-II-214) was calculated for each sample by dividing the RIPC value of one peptide by the RIPC value of its homologous pair peptide to predict the reactivity signature (e.g., I/III vs. II) of each strain type. Ratios of homologous peptides with values 0.95–1.05 were considered inconclusive.

## Results

### Histopathology and molecular characterization

Since the first report of the COUG strain of *T. gondii* in southern sea otters (Cases 1–4) [9], an additional six sea otters with COUG strain infections serving as the primary cause of death have been identified (Cases 5–10). Screening using PCR at the ITS1 locus confirmed the presence of *T. gondii* DNA in brain (n = 4/6), adipose (n = 5/6), and cultured tachyzoites (n = 1/6) in these six newly identified cases. Alignment of MLST sequences derived from adipose (n = 5/6) and cultured tachyzoites (n = 1/6) revealed 100% identity to the COUG strain across all 13 loci for all six cases.

Five cases (Cases 5–9) presented with fatal toxoplasmosis as the primary cause of death similar to that described in the original case series of COUG strain-infected sea otters [9] (Table 1). These five sea otters were all found dead upon stranding in varying states of postmortem decomposition ranging from fresh to severely decomposed. All five cases had grossly visible evidence of steatitis in subcutaneous and internal adipose tissues (epicardial, mesenteric, omental, and perirenal) characterized by yellow or tan to red discoloration and a multinodular, firm to gritty texture (S1 Fig a-b). Adipose tissues were mildly to markedly depleted in all cases. Histologically, affected adipose tissues had widespread granulomatous to lymphoplasmacytic inflammation arranged in dense nodular aggregates. Abundant protozoal organisms consistent with *T. gondii* were present within these inflammatory aggregates, often closely associated with remaining adipocytes (S1 Fig c-d). Lymphoplasmacytic inflammation associated with *T. gondii*-like organisms was present in several additional tissues including brain (n = 5/5), heart (n = 5/5), pancreas (n = 4/5), lungs (n = 3/5), adrenal glands (n = 2/5), diaphragm (n = 1/5), and gastrointestinal smooth muscle (n = 1/5). Similar to previously published cases, *T. gondii*-associated meningoencephalitis was generally mild in these five sea otters suggesting that they died during the acute, systemic phase of infection.

**Table 1:** Stranding data, demographic information, and histopathology results for fatal *Toxoplasma gondii* COUG strain infections in southern sea otters 2020-2024.

Case Number	Stranding Location (County)*	Stranding Date (m/d/y)	Condition at Stranding	Age Class/Sex	Steatitis Severity†	Meningoencephalitis Severity†	Reference
Case 1	SLO	2/18/20	Alive	Adult female	+++	+/++	Miller et al. 2023
Case 2	SLO	2/8/22	Fresh	Adult female	+++	+/++	Miller et al. 2023
Case 3	SC	2/21/22	Moderate decomposition	Immature male	++/+++	+	Miller et al. 2023
Case 4	SLO	3/30/22	Fresh	Adult female	+++	+	Miller et al. 2023
Case 5	SLO	2/20/23	Advanced decomposition	Subadult male	++/+++	+	This study
Case 6	SC	2/27/23	Moderate decomposition	Subadult male	++/+++	+	This study
Case 7	SC	4/15/23	Fresh (frozen)	Subadult male	++/+++	+/++	This study
Case 8	SLO	2/18/24	Fresh	Subadult female	++/+++	+	This study
Case 9	SC	5/13/24	Moderate decomposition	Subadult female	+++	+	This study
Case 10	SLO	11/26/22	Fresh	Adult female	–	++/+++	This study

*SLO = San Luis Obispo County, SC = Santa Cruz County.

† Severity scale: none (-), mild (+), moderate (++), severe (+++).

In contrast, the clinical, gross, and histologic findings observed for the sixth recently identified case (Case 10) were consistent with prior descriptions of chronic toxoplasmosis in sea otters as a primary cause of death (Table 1). Moderate hind limb tremors were observed perimortem and this animal died shortly after stranding. At necropsy, gross lesions characteristic of subacute to chronic systemic protozoal infection were observed including generalized lymphadenomegaly, a distended urinary bladder, and a swollen, wet appearance of the brain. Subcutaneous and internal adipose tissues appeared grossly normal with no evidence of steatitis (S2 Fig a). Histologically, the most significant lesion was moderate to severe lymphoplasmacytic meningoencephalitis with perivascular cuffing and large, multifocal areas of necrosis and mineralization. Abundant protozoal tissue cysts containing bradyzoites morphologically consistent with *T. gondii* were present throughout the brain, often closely associated with inflammation (S2 Fig b). Lymphoplasmacytic inflammation was also present within the heart, although no parasites were seen associated with this inflammation. No significant inflammation or parasites were identified histologically in subcutaneous, omental, mesenteric, epicardial, and perirenal adipose. Minimal inflammation was seen in the pancreas, and no inflammation was observed within gastrointestinal or uterine smooth muscle.

**Fig 1:**
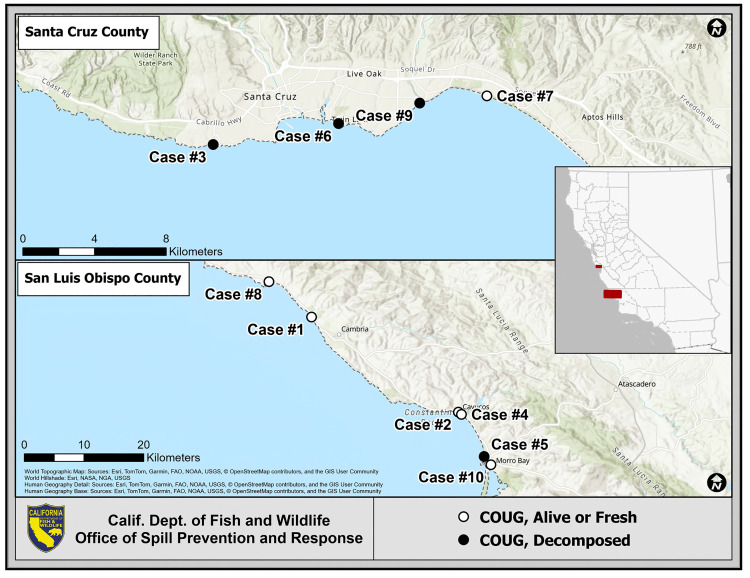
Stranding locations for fatal COUG strain *Toxoplasma gondii* infections in southern sea otters 2020-2024. Map data from © OpenStreetMap (https://www.openstreetmap.org/copyright).

In addition to the previously published cases (three adult females and one immature male), the six newly identified cases consisted of one adult female, one adult male, two subadult females, and two subadult males (Table 1). These six cases had a similar geographic distribution of stranding as the original four cases, with three cases stranding in Santa Cruz County, CA and three stranding in San Luis Obispo County, CA (Table 1, Fig 1). Similar to Cases 1–4, the five cases with grossly and histologically apparent steatitis (Cases 5–9) stranded between February and May in 2023 and 2024, approximately 2–4 weeks after periods of heavy rainfall during the wet season in central coastal California (California Irrigation Management Information System, https://cimis.water.ca.gov/). The only case without gross or histologic evidence of steatitis (Case 10) stranded in November 2022. The heavy rains characteristic of the wet season in California typically do not begin until November; however, unusually heavy rainfall occurred in September 2022 approximately 8–10 weeks prior to the stranding of Case 10, consistent with the more chronic lesions observed in this case and the apparent concentration of parasites and inflammation in the brain.

### RT-PCR for *A. odysseus*

**Fig 2:**
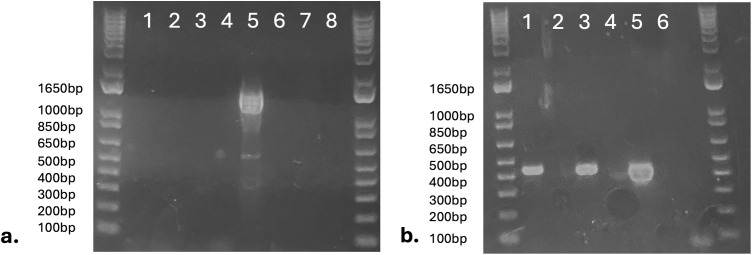
Sea otter-derived COUG strain *Toxoplasma gondii* isolates lack *Apocryptovirus odysseus* RNA compared to TgCgCa1 isolate. (a) RT-PCR targeting *A. odysseus* yielded no amplification for the COUG strain isolates derived from southern sea otters (Case 2 [1 = RT + , 2 = RT-] and Case 10 [3 = RT + , 4 = RT-]), and the negative control *T. gondii* RH strain isolate (7 = RT + , 8 = RT-). A ~ 1341 bp band was produced for the cougar-derived TgCgCa1 COUG strain isolate (5 = RT + , 6 = RT-). (b) RT-PCR targeting *T. gondii* GRA1 yielded appropriately sized (439 bp) bands for Case 2 (1 = RT+), Case 10 (3 = RT+) and RH strain (5 = RT+) samples and no amplification for RT- samples (2, 4, 6). RT + = with reverse transcriptase, RT- = without reverse transcriptase.

The RT-PCR assay designed to amplify the strain of *A. odysseus* previously detected in the cougar-derived TgCgCa1 COUG isolate and a human-derived RUB isolate yielded no amplification in the two sea otter-derived COUG isolates (Cases 2 and 10), nor the RH strain isolate (negative control). A band of the expected size (1341 bp) was amplified for the TgCgCa1 COUG isolate (positive control) (Fig 2a). No amplification occurred for any sample in reactions without reverse transcriptase. Successful amplification of GRA1 cDNA for both sea otter-derived COUG isolates and the RH strain isolate confirmed high quality cDNA synthesis for these samples (Fig 2b).

### Vitamin E testing

Vitamin E levels for the three COUG strain-infected sea otters (Cases 2, 4, and 7) and two sex- and age-matched controls (Controls 1 and 2) are listed in S1 Table. For adult females, sea otters with COUG strain-associated steatitis had higher vitamin E levels than the control animal without clinical, gross, or histologic evidence of steatitis. For subadult males, the sea otter with COUG strain-associated steatitis had a slightly lower vitamin E level than the control animal.

### *Toxoplasma gondii* serotyping

Serum and pericardial fluid samples for all sea otters infected with Type X (n = 9) and Type II (n = 9) strains reacted strongly with the *T. gondii* whole parasite lysate (average OD_405_ > 0.9). Of the COUG strain-infected sea otters for which serum or pericardial fluid was available (n = 7), only those with serum (Case 10) or fresh pericardial fluid (Cases 1, 2, and 4) reacted strongly with the *T. gondii* lysate (average OD_405_ > 0.8). The remaining three COUG strain-infected otters were in a moderate to advanced state of decomposition at the time of necropsy (Cases 5 and 6) or were fresh but the carcass was frozen and thawed prior to necropsy (Case 7). Pericardial fluid samples from these three individuals reacted poorly with the *T. gondii* lysate (average OD_405_ < 0.7); therefore, these three samples were excluded from further evaluation of the ELISA serotyping assay.

**Fig 3:**
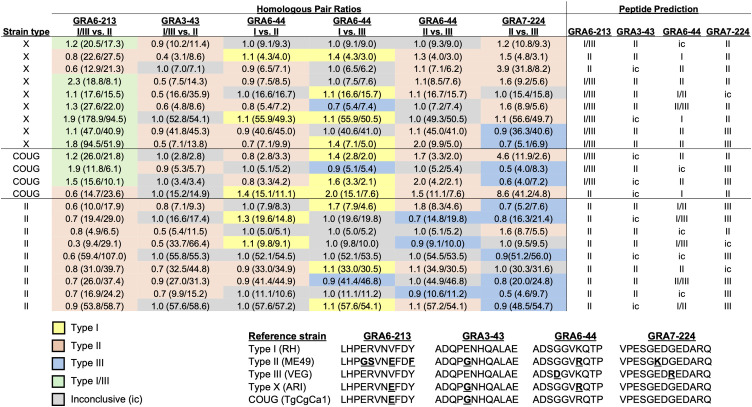
Sea otters infected with different *Toxoplasma gondii* strains exhibit variable reactivity for specific GRA peptides. Ratios were calculated by dividing the relative index percent (RIPC) value for one peptide by the RIPC value of its homologous peptide (in parentheses next to each ratio). Alignments of amino acid sequences for the selected GRA peptides across reference strain types are shown in the lower figure.

**Fig 4:**
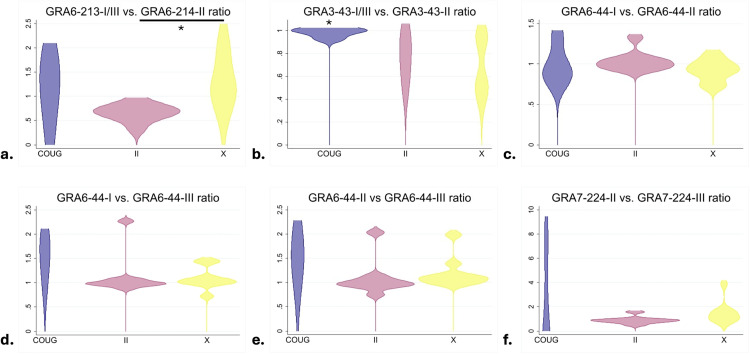
Relative index percent ratios for homologous peptides vary across *Toxoplasma gondii* strains infecting sea otters. (a) A significant difference existed between Type II and Type X relative index percent (RIPC) ratios for GRA6-213-I/III vs. GRA6-214-II (Mann-Whitney test, p = 0.002). (b) The COUG strain had significantly higher RIPC ratios for GRA3-43-I/III vs. GRA3-43-II than Type X and Type II (Mann-Whitney test, p = 0.02, 0.05). (c-f) No significant differences were observed between strains in the RIPC ratios for all other homologous peptide pairs (Kruskal-Wallis test, p > 0.05).

The nine selected peptides were applied to the Type X (n = 9), Type II (n = 9), and COUG strain (n = 4) serum and pericardial fluid samples that exhibited strong reactivity to the lysate. No significant differences in RIPC values were observed between homologous peptide pairs (GRA6-I/III-213 vs. GRA6-II-214, GRA3-I/III-43 vs. GRA3-II-43, GRA7-II-224 vs. GRA7-III-224, and GRA6-I-44 vs. GRA6-II-44 vs. GRA6-III-44) within each strain type (S3 Fig). The ratios of RIPC values between pairs of homologous peptides were calculated to generate a predicted reactivity signature for each sample (Fig 3). Type II samples differed significantly from Type X samples for GRA6-I/III-213 vs. GRA6-II-214 (p = 0.002) (Fig 4a), with this ratio predicting all Type II samples (n = 9/9) as Type II. The majority of Type X samples (n = 7/9) and COUG strain samples (n = 3/4) were predicted as Type I/III with this homologous peptide pair; Type X (ARI) and COUG (TgCgCa1) strains differ from Type I and III strains by a single amino acid in the GRA6–213 peptide sequence (Fig 3). Ratios for GRA3-I/III-43 vs. GRA3-II-43 for COUG strain samples were significantly higher than Type X samples (p = 0.02) and Type II samples (p = 0.05) (Fig 4b). The majority of Type X (n = 7/9) and Type II (n = 6/9) samples were predicted as Type II for this homologous peptide pair. The majority of COUG strain samples (n = 3/4) were inconclusive. Interestingly, the TgCgCa1 COUG strain has an identical amino acid sequence as Types II and X strains for the GRA3–43 peptide (Fig 3). No significant differences between strain types were observed for ratios comparing GRA6-I-44 vs. GRA6-II-44, GRA6-I-44 vs. GRA6-III-44, GRA6-II-44 vs. GRA6-III-44, and GRA7-II-224 vs. GRA4-III-224 (Fig 4c-f).

## Discussion

The COUG strain (TgCgCa1) of *T. gondii* was first discovered in the feces or intestinal contents of two cougars (*Puma concolor*) during a period of increased environmental surveillance for *T. gondii* following a large outbreak of waterborne toxoplasmosis in humans in British Columbia, Canada in 1995 [20, 21]. It is considered to be a rare, atypical North American strain of *T. gondii* that primarily circulates in wildlife [22]. The COUG strain is closely related to other atypical strains shed by wild felids including GUY-2004-JAG1 isolated from a jaguar in French Guiana and TgPumaMe1 isolated from a puma in Mexico, which also exhibits high virulence in mice [23,24]. The first report of the COUG strain in southern sea otters in 2023 raised alarm as this seemingly virulent strain appeared to be capable of causing rapid death in otherwise healthy animals. Identification of the COUG strain in additional sea otters with fatal toxoplasmosis in subsequent years indicates that this pathogen continues to be an emerging threat to federally protected southern sea otters, whose population is still struggling to return to its historic size and range due to numerous diseases and ecological factors.

The six newly identified cases of COUG strain infections in sea otters that died in 2022–2024 followed similar spatiotemporal patterns as the previously published cases. Unlike toxoplasmosis caused by other strains which do not exhibit clear seasonal patterns in sea otters [1], COUG strain infections appear to have a strong association with the winter and spring rainy season in the central coast of California. Increased freshwater runoff following precipitation events likely facilitates transport of numerous infective oocysts from terrestrial habitats into the marine environment. Sea otters may become infected by consuming sporulated oocysts that concentrate in invertebrate prey items such as snails and mussels [25,26]. All cases occurred shortly after periods of heavy rainfall during the peak wet season (Cases 1–9) or after unusual heavy precipitation in the fall (Case 10). The strong seasonal pattern for COUG strain infections in sea otters likely reflects infected sea otters dying acutely post-infection as evidenced by the abundant parasites within the lungs and associated interstitial pneumonia, while other *T. gondii* strains typically cause death in the subacute or chronic stages post-infection where lesions are focused on the central nervous system [5].

The six recently identified cases stranded in similar locations as the previously reported cases, with a total of four cases stranding within Monterey Bay in Santa Cruz County near the northern end of the sea otter range, and the remaining six cases stranding within Estero Bay in San Luis Obispo County near the southern end of the sea otter range (Fig 1). The geographically separate distribution of cases suggests that two distinct point sources exist for introduction of the COUG strain into the marine environment. However, the stranding location may not necessarily equate to the site of initial infection, as male sea otters can migrate hundreds of kilometers [27], and carcasses can drift along ocean currents from the original site of infection or death. Much of the southern sea otter range between Monterey Bay and Estero Bay is inaccessible to humans which limits the sighting and recovery of live-stranded animals and carcasses in this region. The terrain in this region is also less likely to retain animals and carcasses that wash ashore, further limiting detection and recovery efforts. It is possible that infection and mortality attributed to the COUG strain occurs throughout the southern sea otter range but has only been identified in Monterey Bay and Estero Bay to-date due to increased human presence and feasibility of animal and carcass recovery in these areas.

The two apparent high-risk areas for COUG strain infections in sea otters may reflect unique characteristics of the felid populations shedding oocysts into local watersheds at both sites. Previous studies characterizing *T. gondii* strains from wild and domestic felids along the central coast of California have described the presence of Type I alleles at the B1 locus from tissue or fecal samples collected near Monterey Bay and San Luis Obispo County, but these samples could not be fully genotyped due to low quality/quantity of parasite DNA [28,29]. The COUG strain similarly has a Type I allele at the B1 locus, so it is possible that these samples from domestic cats or wild felids represented COUG strain-infected definitive hosts present in terrestrial habitats near known high risk areas for COUG strain infections in sea otters. Additional studies are needed to fully characterize the strains present in wild and domestic felids to assess whether these definitive hosts are shedding COUG strain oocysts into coastal watersheds.

While three of the four initially reported COUG-infected sea otter cases were adult females, the six newly identified cases comprised mixed age classes and sexes. No strong age- or sex-related associations with fatal toxoplasmosis have been reported for other *T. gondii* strains infecting sea otters [1, 6]. The ability of the COUG strain to cause acute death in healthy adult sea otters highlights the virulent nature of this atypical parasite strain in this host species.

Of 10 confirmed COUG strain infections in sea otters to date, nine individuals died due to acute, systemic toxoplasmosis characterized by severe steatitis of all internal and subcutaneous adipose tissues accompanied by relatively mild meningoencephalitis. In contrast, one COUG strain-infected sea otter died from severe *T. gondii*-associated meningoencephalitis with no gross or microscopic evidence of steatitis. This suggests that some sea otters may survive the acute effects of COUG strain infection but later succumb to the central nervous system-centered lesions typical of chronic toxoplasmosis. The pathophysiologic mechanisms driving the affinity of the COUG strain for adipose tissue currently remain unknown. Host lipid reserves play an important role in intracellular survival for *T. gondii* [30]. The degree of parasitism within adipose tissue appears to be especially high for the COUG strain in infected sea otters, and it is possible that enhanced utilization of host lipids compared to other strains could play a role in the pathogenesis of COUG strain-associated steatitis.

Other host factors or comorbidities could also influence if and how COUG strain-infected sea otters develop steatitis. No published reference values exist for hepatic vitamin E levels in sea otters, but the results of this study do not suggest vitamin E deficiency is a contributor to steatitis in otters infected with the COUG strain. However, our sample size was limited to three COUG-infected sea otters and two age- and sex-matched control animals. Larger scale investigations of liver or plasma concentrations of vitamin E are needed to confirm or rule out any potential contribution of vitamin E deficiency in the pathogenesis of steatitis in sea otters.

The parasite-infecting narnavirus *A. odysseus* that was previously identified in the cougar-derived TgCgCa1 COUG strain isolate [10] was not detected in the COUG strain isolates obtained from sea otter brains in this study. The TgCgCa1 isolate and the two sea otter-derived isolates were separated spatially by hundreds of miles (British Columbia, Canada vs. California, USA) and temporally by over 20 years, during which time the TgCgCa1 isolate has been maintained in cell culture through several passages. The definitive hosts shedding COUG strain oocysts that ultimately infected these two sea otters also remain unknown. Differences in the geographic origin and date of isolation, the number of cell culture passages, and potentially the definitive host sources may account for the lack of *A. odysseus* detected in the COUG strain isolates obtained from sea otters. Although the sea otter-derived isolates were identical to TgCgCa1 at the 13 loci evaluated using MLST, it is possible that further characterization at additional loci could reveal polymorphisms that would suggest that the sea otter-derived isolates represent a closely related, COUG-like strain. This possible divergence may also account for the lack of *A. odysseus* detection in the sea otter-derived isolates. The role this virus plays in the pathogenesis of naturally occurring COUG strain infections remains uncertain, and screening additional sea otter-derived COUG isolates in the future may elucidate this role.

Development of a serotyping tool to determine the strain of *T. gondii* infecting sea otters using serum collected from live-stranded animals would provide an important clinical diagnostic advancement, as current methods to determine *T. gondii* strain type rely on molecular characterization techniques (restriction fragment length polymorphism [RFLP], MLST, or microsatellite typing) that require greater time and expense, and a sufficient amount and quality of DNA derived from tissues collected at postmortem examination. Our study results suggest that Type X strains can be distinguished from Type II strains using the ratio of GRA6-I/III-213 vs. GRA6-II-214, with Type X strains predicted to be Type I/III and Type II strains predicted to be Type II. Although not significant with the available data, the COUG strain also appeared to trend towards Type I/III with this homologous peptide pair similar to Type X strains. Samples available from COUG strain-infected sea otters were limited (n = 4), and it is possible that a significant difference may emerge between COUG and Type II strains with a larger sample size. The ability of GRA6-I/III-213 vs. GRA6-II-214 ratios to distinguish Type II strains from Type X strains, and possibly the COUG strain, has clinical value as only Type X and COUG strains have been identified in cases of fatal toxoplasmosis in sea otters, whereas Type II strain infections appear to be sublethal [6,9]. The COUG strain differed significantly from Type II and X strains for the GRA3-I/III-43 vs. GRA3-II-43 ratio; however, reference sequences of these strains (ME49, ARI, TgCgCa1) have identical GRA3–43 peptide sequences. The greater ratios and predicted reactivity for the COUG strain samples may be due to polymorphisms in the GRA3 peptide sequence unique to sea otter-derived COUG isolates compared to the TgCgCa1 isolate or represent spurious results due to the small number of samples tested. Given the inconclusive prediction for many of the COUG strain samples, it is also possible that the COUG strain has other immunogenic epitopes on the GRA3 protein or other proteins that play a more important role in the immune response than the selected GRA3–43 peptide. Evaluation of a larger sample set will help to clarify the reactivity profiles of different strains across homologous peptide pairs and validate these assays for clinical diagnostic use in sea otters and potentially other susceptible hosts.

The COUG strain of *T. gondii* is an emerging threat to the southern sea otter population, causing sea otter mortalities nearly every year since 2020. Much remains unknown about the origin, pathogenesis, and long-term population health impacts of this pathogen in sea otters. Additional studies exploring the *T. gondii* strains circulating in local felid populations and those present within the terrestrial and marine environments may provide insight on the origins of this pathogen and how and where sea otters become exposed. Understanding the lesions associated with infection with the COUG strain and developing tools to identify and characterize these infections will expedite disease diagnosis and optimize clinical management of live-stranded sea otters. Continued surveillance for the COUG strain in coastal environments may also serve to identify additional susceptible hosts that share habitats and resources with sea otters, including other marine mammals and humans.

## Supporting information

S1 Fig Gross and histopathologic lesions of *Toxoplasma gondii* COUG strain-infected sea otters with steatitis. (a-b) In five of the newly identified cases of southern sea otters infected with the COUG strain of *T. gondii* (Cases 5–9), grossly apparent steatitis was present characterized by red-yellow discoloration and a multinodular appearance of subcutaneous (Case 7) and internal adipose tissue including the mesentery (Case 5). (c-d) These lesions corresponded histologically with dense nodular aggregates of granulomatous inflammation with abundant intralesional *T. gondii* organisms (arrows) often closely associated with remnant adipocytes (stain: hematoxylin and eosin, H&E).
(TIF)

S2 Fig Gross and histopathologic lesions of *Toxoplasma gondii* COUG strain-infected sea otter with meningoencephalitis that lacked steatitis. (a) Normal subcutaneous adipose tissue in a southern sea otter infected with the COUG strain of *Toxoplasma gondii* (Case 10) that had no gross or histologic evidence of protozoal-associated steatitis in subcutaneous or internal adipose tissues. (b) This individual died due to severe lymphoplasmacytic meningoencephalitis with abundant large, intralesional *T. gondii* tissue cysts (inset) (stain: hematoxylin and eosin, H&E).
(TIF)

S1 Table Hepatic vitamin E concentrations in COUG strain-infected and uninfected sea otters. Vitamin E concentrations were compared between COUG strain-infected sea otters with gross and histologically confirmed protozoal steatitis (Cases 2, 4, and 7) and age- and sex-matched control sea otters lacking gross and histologic evidence of steatitis (Controls 1 and 2).
(DOCX)

S3 Fig Distribution of relative index percent (RIPC) values for GRA peptides by strain type. (a-c) No significant differences in RIPC values were observed between homologous GRA peptide pairs within the COUG strain (a), Type II strain (b), or Type X strain (c) (Mann-Whitney test, p > 0.05).
(TIF)

S1 File Raw images. (PDF)

## References

[1] Miller MA, Moriarty ME, Henkel L, Tinker MT, Burgess TL, Batac FI, et al. Predators, Disease, and Environmental Change in the Nearshore Ecosystem: Mortality in Southern Sea Otters (*Enhydra lutris nereis*) From 1998–2012. Front Mar Sci. 2020;7. doi: 10.3389/fmars.2020.00582

[2] Thomas NJ, Dubey JP, Lindsay DS, Cole RA, Meteyer CU. Protozoal meningoencephalitis in sea otters (*Enhydra lutris*): a histopathological and immunohistochemical study of naturally occurring cases. J Comp Pathol. 2007;137(2–3):102–21. doi: 10.1016/j.jcpa.2007.05.001 17692867

[3] Dubey JP, Frenkel JK. Cyst-induced toxoplasmosis in cats. J Protozool. 1972;19(1):155–77. doi: 10.1111/j.1550-7408.1972.tb03431.x 5008846

[4] Hutchison WM, Dunachie JF, Siim JC, Work K. Life cycle of *Toxoplasma gondii*. Br Med J. 1969;4(5686):806. doi: 10.1136/bmj.4.5686.806-b 5359949 PMC1630290

[5] Miller M, Shapiro K, Murray MJ, Haulena M, Raverty S. Protozoan Parasites of Marine Mammals. CRC Handbook of Marine Mammal Medicine. 3rd ed. Boca Raton, FL: CRC Press Taylor & Francis Group. 2018. p. 425–69.

[6] Shapiro K, VanWormer E, Packham A, Dodd E, Conrad PA, Miller M. Type X strains of *Toxoplasma gondii* are virulent for southern sea otters (*Enhydra lutris nereis*) and present in felids from nearby watersheds. Proc Biol Sci. 2019;286(1909):20191334. doi: 10.1098/rspb.2019.1334 31431162 PMC6732395

[7] Khan A, Taylor S, Ajioka JW, Rosenthal BM, Sibley LD. Selection at a single locus leads to widespread expansion of *Toxoplasma gondii* lineages that are virulent in mice. PLoS Genet. 2009;5(3):e1000404. doi: 10.1371/journal.pgen.1000404 19266027 PMC2644818

[8] Melo MB, Nguyen QP, Cordeiro C, Hassan MA, Yang N, McKell R, et al. Transcriptional analysis of murine macrophages infected with different *Toxoplasma* strains identifies novel regulation of host signaling pathways. PLoS Pathog. 2013;9(12):e1003779. doi: 10.1371/journal.ppat.1003779 24367253 PMC3868521

[9] Miller MA, Newberry CA, Sinnott DM, Batac FI, Greenwald K, Reed A, et al. Newly detected, virulent *Toxoplasma gondii* COUG strain causing fatal steatitis and toxoplasmosis in southern sea otters (*Enhydra lutris nereis*). Front Mar Sci. 2023;10. doi: 10.3389/fmars.2023.1116899

[10] Gupta P, Hiller A, Chowdhury J, Lim D, Lim DY, Saeij JPJ, et al. A parasite odyssey: An RNA virus concealed in *Toxoplasma gondii*. Virus Evol. 2024;10(1):veae040. doi: 10.1093/ve/veae040 38817668 PMC11137675

[11] Williams BH, Burek Huntington K, Miller M. Mustelids. Pathol Wildlife Zoo Animals. Elsevier. 2018. p. 287–304. doi: 10.1016/b978-0-12-805306-5.00011-0

[12] Arranz-Solís D, Cordeiro C, Young LH, Dardé ML, Commodaro AG, Grigg ME, et al. Serotyping of *Toxoplasma gondii* infection using peptide membrane arrays. Front Cell Infect Microbiol. 2019;9:408. doi: 10.3389/fcimb.2019.00408 31850240 PMC6895565

[13] Arranz-Solís D, Carvalheiro CG, Zhang ER, Grigg ME, Saeij JPJ. *Toxoplasma* GRA Peptide-Specific Serologic Fingerprints Discriminate Among Major Strains Causing Toxoplasmosis. Front Cell Infect Microbiol. 2021;11:621738. doi: 10.3389/fcimb.2021.621738 33680990 PMC7935526

[14] Arranz-Solís D, Tana LR, Tejerina-de-Uribe E, López-Ureña NM, Koudela B, Francia ME, et al. A combination of GRA3, GRA6 and GRA7 peptides offer a useful tool for serotyping type II and III *Toxoplasma gondii* infections in sheep and pigs. Front Cell Infect Microbiol. 2024;14:1384393. doi: 10.3389/fcimb.2024.1384393 38720960 PMC11076764

[15] Rejmanek D, Vanwormer E, Miller MA, Mazet JAK, Nichelason AE, Melli AC, et al. Prevalence and risk factors associated with Sarcocystis neurona infections in opossums (*Didelphis virginiana*) from central California. Vet Parasitol. 2009;166(1–2):8–14. doi: 10.1016/j.vetpar.2009.08.013 19735983

[16] Grigg ME, Boothroyd JC. Rapid identification of virulent type I strains of the protozoan pathogen *Toxoplasma gondii* by PCR-restriction fragment length polymorphism analysis at the B1 gene. J Clin Microbiol. 2001;39(1):398–400. doi: 10.1128/JCM.39.1.398-400.2001 11136812 PMC87743

[17] Su C, Khan A, Zhou P, Majumdar D, Ajzenberg D, Dardé M-L, et al. Globally diverse *Toxoplasma gondii* isolates comprise six major clades originating from a small number of distinct ancestral lineages. Proc Natl Acad Sci U S A. 2012;109(15):5844–9. doi: 10.1073/pnas.1203190109 22431627 PMC3326454

[18] Kong J-T, Grigg ME, Uyetake L, Parmley S, Boothroyd JC. Serotyping of *Toxoplasma gondii* infections in humans using synthetic peptides. J Infect Dis. 2003;187(9):1484–95. doi: 10.1086/374647 12717631

[19] Miller MA, Gardner IA, Packham A, Mazet JK, Hanni KD, Jessup D, et al. Evaluation of an indirect fluorescent antibody test (ifat) for demonstration of antibodies to *Toxoplasma gond*ii in the sea otter (*Enhydra lutris*). J Parasitol. 2002;88(3):594–9. doi: 10.1645/0022-3395(2002)088[0594:eoaifa]2.0.co;2 12099433

[20] Aramini JJ, Stephen C, Dubey JP, Engelstoft C, Schwantje H, Ribble CS. Potential contamination of drinking water with Toxoplasma gondii oocysts. Epidemiol Infect. 1999;122(2):305–15. doi: 10.1017/s0950268899002113 10355797 PMC2809621

[21] Dubey JP, Quirk T, Pittt JA, Sundar N, Velmurugan GV, Kwok OCH, et al. Isolation and genetic characterization of *Toxoplasma gondii* from raccoons (*Procyon lotor*), cats (*Felis domesticus*), striped skunk (*Mephitis mephitis*), black bear (*Ursus americanus*), and cougar (*Puma concolor*) from Canada. J Parasitol. 2008;94(1):42–5. doi: 10.1645/GE-1349.1 18372620

[22] Lorenzi H, Khan A, Behnke MS, Namasivayam S, Swapna LS, Hadjithomas M, et al. Local admixture of amplified and diversified secreted pathogenesis determinants shapes mosaic *Toxoplasma gondii* genomes. Nat Commun. 2016;7:10147. doi: 10.1038/ncomms10147 26738725 PMC4729833

[23] Mercier A, Ajzenberg D, Devillard S, Demar MP, de Thoisy B, Bonnabau H, et al. Human impact on genetic diversity of *Toxoplasma gondii*: example of the anthropized environment from French Guiana. Infect Genet Evol. 2011;11(6):1378–87. doi: 10.1016/j.meegid.2011.05.003 21600306

[24] Dubey JP, Alvarado-Esquivel C, Herrera-Valenzuela VH, Ortiz-Diaz JJ, Oliveira S, Verma SK, et al. A new atypical genotype mouse virulent strain of *Toxoplasma gondii* isolated from the heart of a wild caught puma (*Felis concolor*) from Durango, Mexico. Veterinary Parasitol. 2013;197(3–4):674–7. doi: 10.1016/j.vetpar.2013.06.005 23849518

[25] Johnson CK, Tinker MT, Estes JA, Conrad PA, Staedler M, Miller MA, et al. Prey choice and habitat use drive sea otter pathogen exposure in a resource-limited coastal system. Proc Natl Acad Sci U S A. 2009;106(7):2242–7. doi: 10.1073/pnas.0806449106 19164513 PMC2650139

[26] Miller MA, Miller WA, Conrad PA, James ER, Melli AC, Leutenegger CM, et al. Type X *Toxoplasma gondii* in a wild mussel and terrestrial carnivores from coastal California: new linkages between terrestrial mammals, runoff and toxoplasmosis of sea otters. Int J Parasitol. 2008;38(11):1319–28. doi: 10.1016/j.ijpara.2008.02.005 18452923

[27] Tinker MT, Tomoleoni J, LaRoche N, Bowen L, Miles AK, Murray M, et al. Southern sea otter range expansion and habitat use in the Santa Barbara Channel, California. Open-File Report. US Geological Survey. 2017. doi: 10.3133/ofr20171001

[28] Zhu S, Camp L, Patel A, VanWormer E, Shapiro K. High prevalence and diversity of *Toxoplasma gondii* DNA in feral cat feces from coastal California. PLoS Negl Trop Dis. 2023;17(12):e0011829. doi: 10.1371/journal.pntd.0011829 38100522 PMC10756541

[29] VanWormer E, Miller MA, Conrad PA, Grigg ME, Rejmanek D, Carpenter TE, et al. Using molecular epidemiology to track *Toxoplasma gondii* from terrestrial carnivores to marine hosts: implications for public health and conservation. PLoS Negl Trop Dis. 2014;8(5):e2852. doi: 10.1371/journal.pntd.0002852 24874796 PMC4038486

[30] Nolan SJ, Romano JD, Coppens I. Host lipid droplets: an important source of lipids salvaged by the intracellular parasite *Toxoplasma gondii*. PLoS Pathog. 2017;13(6):e1006362. doi: 10.1371/journal.ppat.1006362 28570716 PMC5469497

