# Supplementary figures and images for: Fatal Toxoplasma gondii COUG strain infections in southern sea otters (Enhydra lutris nereis): New insight on contributing factors and parasite serotyping
Source: PLoS One. 2025 Sep 10;20(9):e0332223. doi: 10.1371/journal.pone.0332223 (PMC12422482; doi:10.1371/journal.pone.0332223)

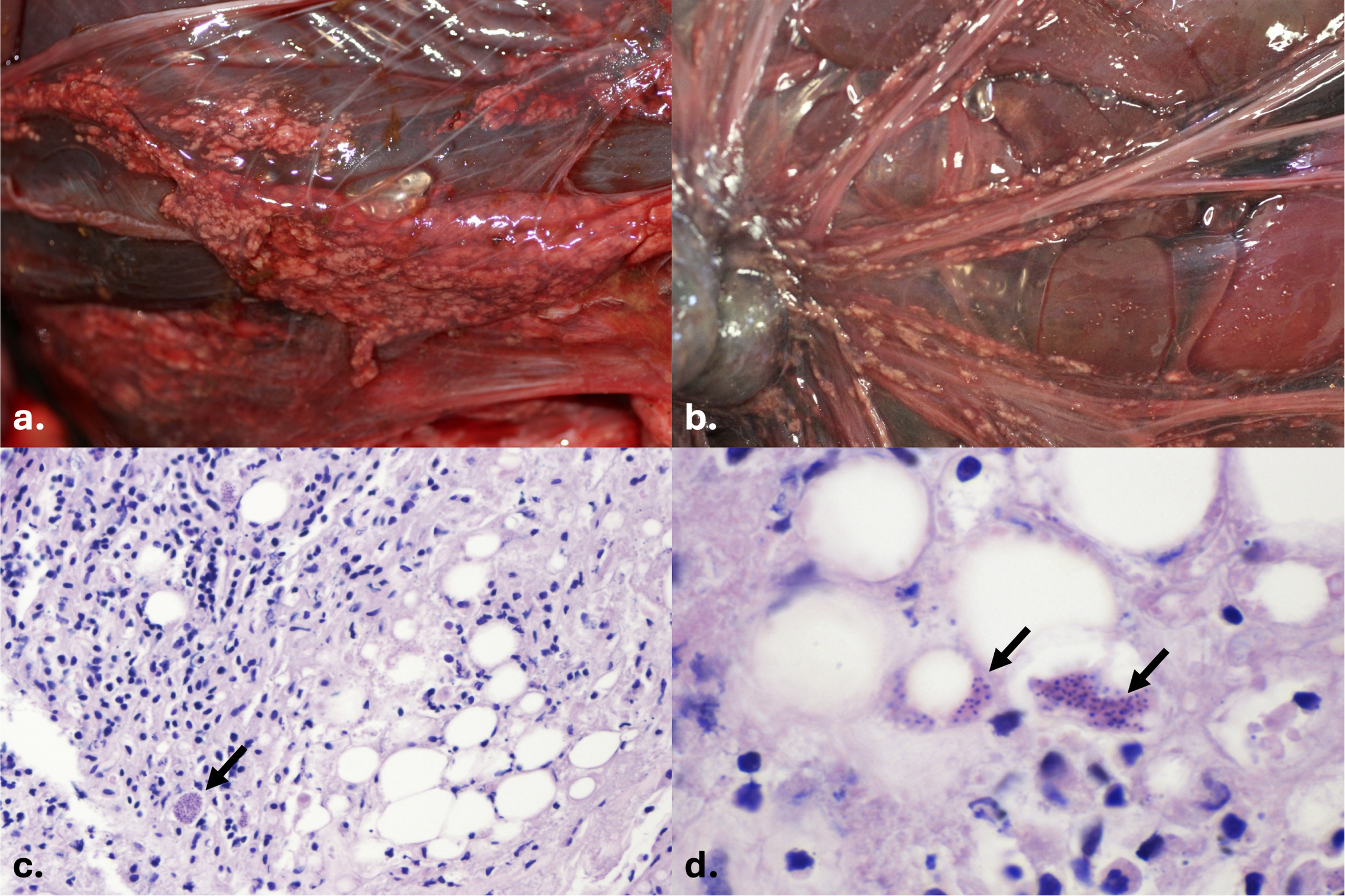

Supplement: S1 Fig — (a-b) In five of the newly identified cases of southern sea otters infected with the COUG strain of T. gondii (Cases 5–9), grossly apparent steatitis was present characterized by red-yellow discoloration and a multinodular appearance of subcutaneous (Case 7) and internal adipose tissue including the mesentery (Case 5). (c-d) These lesions corresponded histologically with dense nodular aggregates of granulomatous inflammation with abundant intralesional T. gondii organisms (arrows) often closely associated with remnant adipocytes (stain: hematoxylin and eosin, H&E). (TIF) [file pone.0332223.s001.tif]

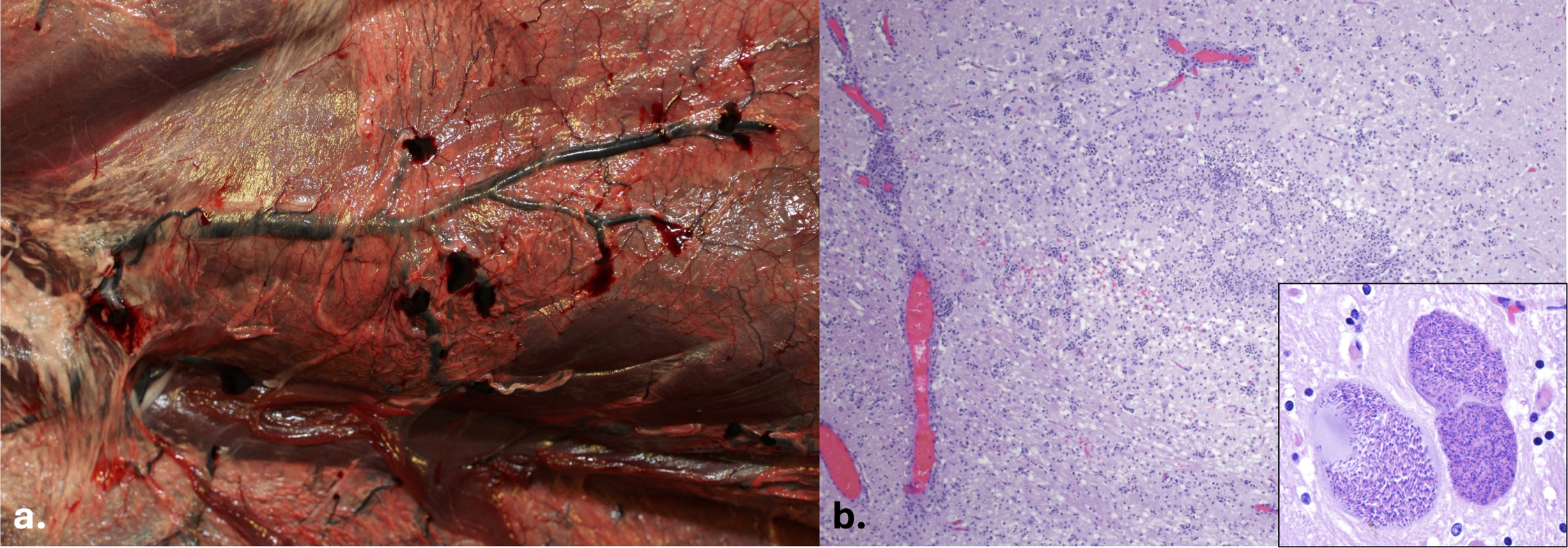

Supplement: S2 Fig — (a) Normal subcutaneous adipose tissue in a southern sea otter infected with the COUG strain of Toxoplasma gondii (Case 10) that had no gross or histologic evidence of protozoal-associated steatitis in subcutaneous or internal adipose tissues. (b) This individual died due to severe lymphoplasmacytic meningoencephalitis with abundant large, intralesional T. gondii tissue cysts (inset) (stain: hematoxylin and eosin, H&E). (TIF) [file pone.0332223.s002.tif]

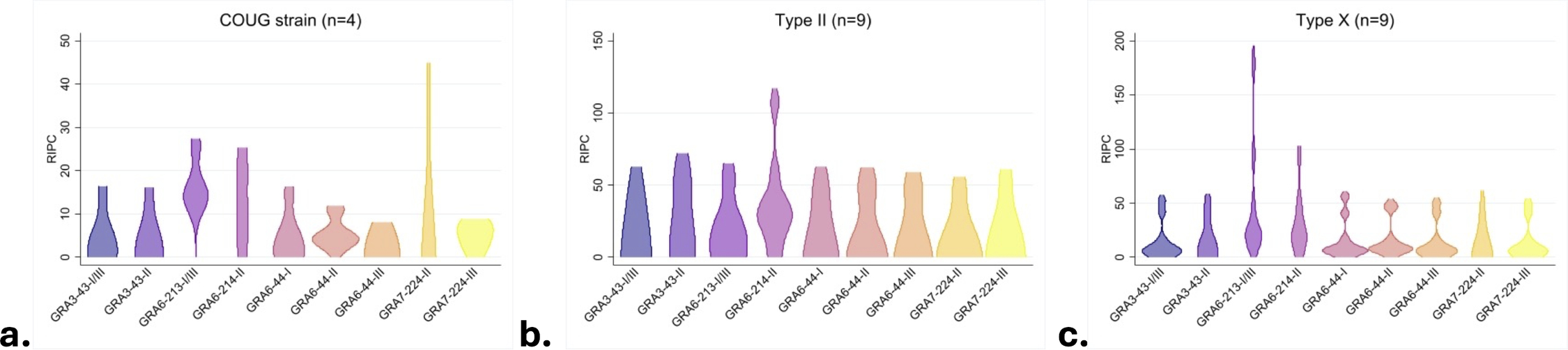

Supplement: S3 Fig — (a-c) No significant differences in RIPC values were observed between homologous GRA peptide pairs within the COUG strain (a), Type II strain (b), or Type X strain (c) (Mann-Whitney test, p > 0.05). (TIF) [file pone.0332223.s004.tif]
