# Supplementary material for: Fatal Toxoplasma gondii COUG strain infections in southern sea otters (Enhydra lutris nereis): New insight on contributing factors and parasite serotyping
Source: PLoS One. 2025 Sep 10;20(9):e0332223. doi: 10.1371/journal.pone.0332223 (PMC12422482; doi:10.1371/journal.pone.0332223)
Supplement: S1 Table — Vitamin E concentrations were compared between COUG strain-infected sea otters with gross and histologically confirmed protozoal steatitis (Cases 2, 4, and 7) and age- and sex-matched control sea otters lacking gross and histologic evidence of steatitis (Controls 1 and 2). (DOCX) [file pone.0332223.s003.docx]

**S1 Table. Hepatic vitamin E concentrations in COUG strain-infected and uninfected sea otters.**

Vitamin E concentrations were compared between COUG strain-infected sea otters with gross and histologically confirmed protozoal steatitis (Cases 2, 4, and 7) and age- and sex-matched control sea otters lacking gross and histologic evidence of steatitis (Controls 1 and 2).

|  | **Age class/Sex** | **Vitamin E concentrations (ppm)** |
| --- | --- | --- |
| Case 2 | Adult female | 18 |
| Case 4 | Adult female | 21 |
| Control 1 | Adult female | 13 |
| Case 7 | Subadult male | 19 |
| Control 2 | Subadult male | 27 |
