# Supplementary material for: Fatal Toxoplasma gondii COUG strain infections in southern sea otters (Enhydra lutris nereis): New insight on contributing factors and parasite serotyping
Source: PLoS One. 2025 Sep 10;20(9):e0332223. doi: 10.1371/journal.pone.0332223 (PMC12422482; doi:10.1371/journal.pone.0332223)
Supplement: S1 File — (PDF) [file pone.0332223.s005.pdf]

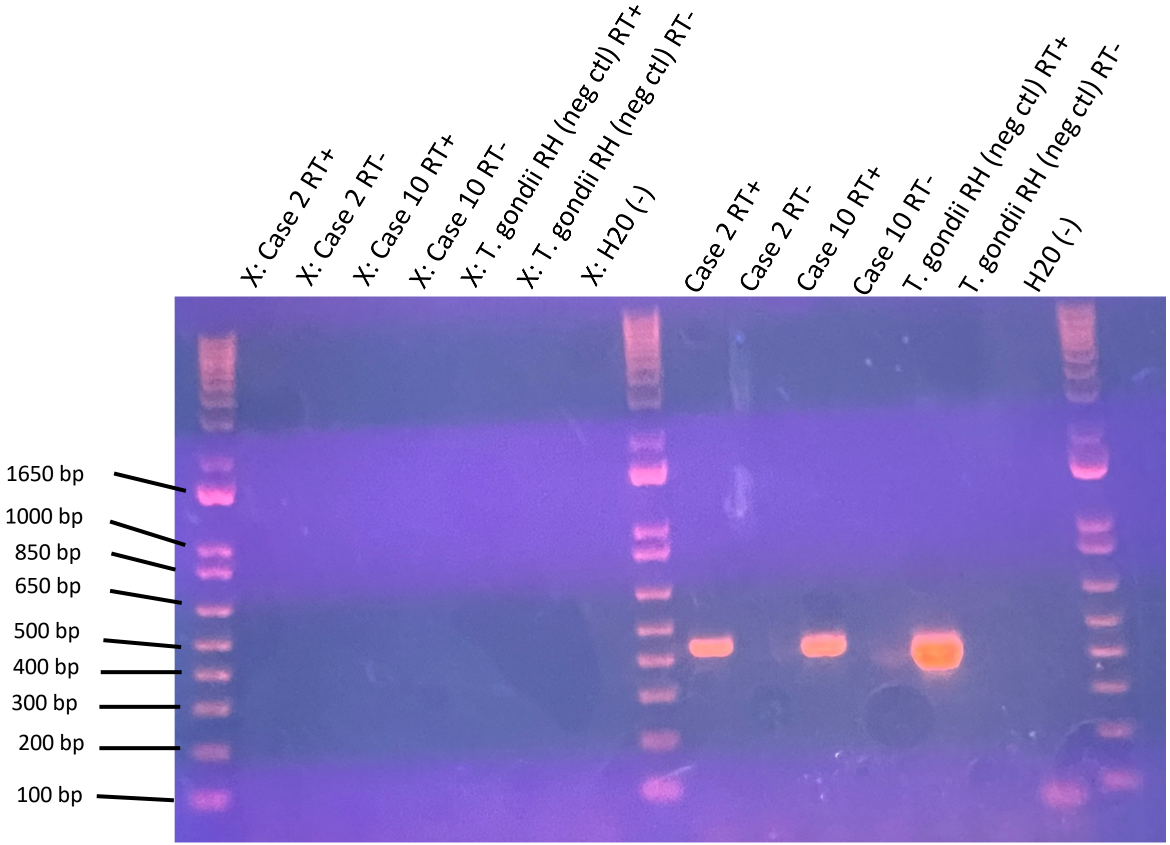

X: NarnaBoth FW1 & RV1 – 1341bp, initial gel not included because failed to include positive control, UV transilluminator

Fig 2B) GRA1 FW1 & RV1 – 439bp, UV transilluminator

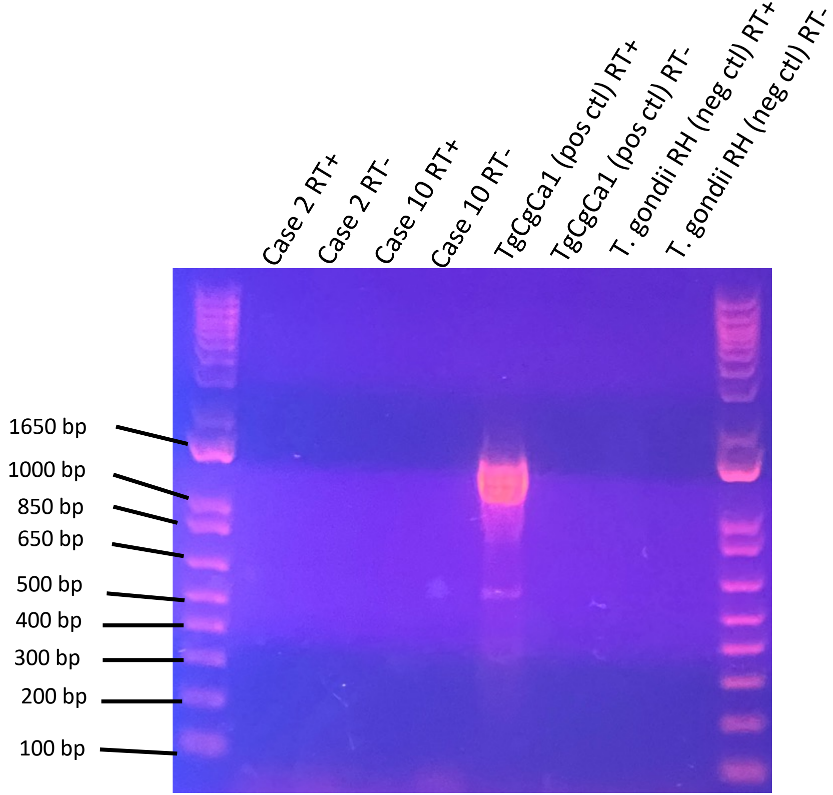

Fig 2A) NarnaBoth FW1 & RV1 – 1341bp (repeated due to no positive control in initial gel), UV transilluminator
